# Laparoscopic-assisted colectomy in a patient with colon cancer after percutaneous endoscopic gastrostomy

**DOI:** 10.1186/1477-7819-10-116

**Published:** 2012-06-22

**Authors:** Hirotoshi Kobayashi, Tetsuro Higuchi, Hiroyuki Uetake, Satoru Iida, Toshiaki Ishikawa, Kenichi Sugihara

**Affiliations:** 1 Center for Minimally Invasive Surgery, Division of Colorectal Surgery, Tokyo Medical and Dental University, 1-5-45 Yushima, Bunkyo-ku, Tokyo, 113-8519, Japan; 2 Department of Surgical Oncology, Tokyo Medical and Dental University, Tokyo, Japan; 3 Department of Translational Oncology, Tokyo Medical and Dental University, Tokyo, Japan

**Keywords:** Colon cancer, Laparoscopic colectomy, Percutaneous endoscopic gastrostomy

## Abstract

**Background:**

A number of patients undergo percutaneous endoscopic gastrostomy (PEG) under various conditions. Open colectomy is usually performed for colon cancer in patients with PEG because the safety of the laparoscopic approach for such patients has not been established. However, if the laparoscopic approach is possible in patients with PEG, it will be less invasive and more helpful in rehabilitation into society.

**Case presentation:**

We describe the case of a 64-year-old male with a T1 adenocarcinoma of the ascending colon 2 years after surgery for nasal cancer and PEG for dysphagia. The patient did not have any distant metastases or malignant tumors on preoperative computed tomography and positron-emission tomography. He underwent laparoscopic-assisted colectomy (LAC) with lymph node dissection. No complications developed during or after the surgery.

**Conclusions:**

LAC could be a potential option for the treatment of colon cancer in patients who have undergone PEG. To our knowledge, this is the first recorded case of an ascending colon cancer treated with LAC under the condition of gastrostoma.

## Background

Colorectal cancer is one of the diseases increasing the most in Japan [1,2]. The most promising treatment for colorectal cancer is curative resection. Since laparoscopic-assisted colectomy (LAC) was reported in 1991 [3], LAC has prevailed as the treatment for colorectal cancer.

Japan is rapidly aging. Percutaneous endoscopic gastrostomy (PEG) has been performed to improve the nutrition of a number of patients for various conditions. Since endoscopic insertion of a gastrostomy tube was first described in 1980 [4], various instruments and techniques have been developed. The number of colorectal cancers arising in patients with PEG is expected to increase.

We present the case of an early invasive carcinoma of the ascending colon that was treated with LAC. The patient had previously undergone PEG for dysphagia after surgical treatment for nasal cancer. To our knowledge, this is the first recorded case of an ascending colon cancer treated with LAC under the condition of gastrostoma.

## Case presentation

A 64-year-old male was diagnosed with an early invasive adenocarcinoma of the ascending colon 2 years after the surgery for nasal cancer. His face was reconstructed using the flap of the abdominal rectus muscle, and PEG was performed for the dysphagia. The tumor in the ascending colon was a 20-mm sessile polyp, and the depth of tumor invasion was diagnosed as T1 using colonoscopy. A full metastatic workup was performed including chest x-ray and computed tomography. These examinations did not demonstrate any evidence of metastatic disease. In addition, there was no evidence of other malignant tumors by positron-emission tomography. Tumor markers, including serum carcinoembryonic antigen and CA 19–9, were not elevated. The patient’s performance status according to the Eastern Cooperative Oncology Group was 1 at that time.

**Figure 1:**
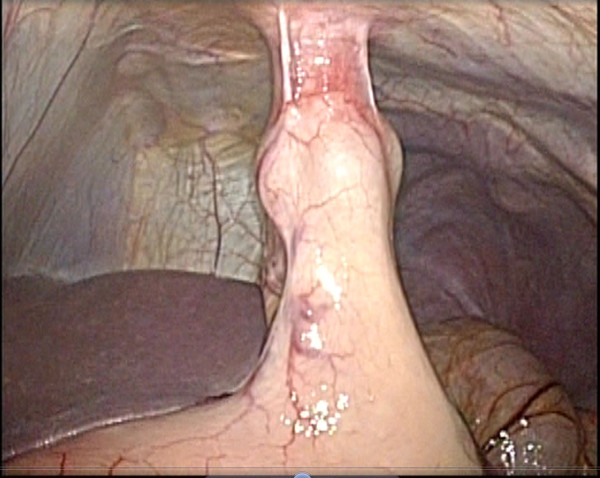
**After insufflation of CO**_**2**_**gas, the abdominal cavity was observed.** The fistula between the abdominal wall and the stomach was robust.

Right hemicolectomy with lymph node dissection was carried out laparoscopically. At the start of LAC, the insufflation pressure was gradually increased and was fixed at 10 mmHg after the confirmation of safety in the gastrostoma (Figure 1). There was no adhesion in the abdominal cavity. In addition to the camera port, one 12-mm and three 5-mm trocars were inserted. The gastrostoma did not prevent any of the steps of the LAC procedure. The ileocecal and right colic arteries were ligated and cut at the roots. The hepatic flexure takedown was easy because the stomach was hung up. After removal of the specimen, functional end-to-end anastomosis was performed.

Pathological examination showed a well-differentiated adenocarcinoma invading the submucosa, which had neither lymphatic nor venous invasion. The depth of submucosal invasion was 10 mm. The 36 lymph nodes retrieved had no cancer cells.

The patient did well postoperatively and was discharged home on postoperative day 9. He remains in good health 2 years after LAC.

## Discussion

A number of patients undergo PEG for the various reasons. Although various enteral feeding techniques exist, PEG is more comfortable for patients than using a nasogastric tube, and is less invasive than open or laparoscopic gastrostomy. More than approximately 100,000 PEG procedures per year are performed in the USA [5].

Some patients with long-term gastrostomy may develop colorectal cancer. In the present case, an early invasive cancer of the ascending colon was found after surgery for nasal cancer. In addition, a gastrostomy for dysphagia was present. Although curative resection for the ascending colon cancer was planned, the problem was choosing the colectomy method, that is, open or laparoscopic. LAC is recommended as a standard procedure for an early invasive colorectal cancer according to the Japanese Society for Cancer of the Colon and Rectum (JSCCR) Guidelines for the Treatment of Colorectal Cancer [6]. However, there was no evidence regarding the safety of LAC in patients after PEG. Although we searched PubMed using the key words ‘laparoscopic colectomy,’ ‘colorectal cancer,’ and ‘percutaneous endoscopic gastrostomy,’ there were no matching results. In the present case, the PEG procedure had been performed 2 years ago. The fistula between the stomach and abdominal wall was considered to be well built. The PEG tract is usually considered to be mature if 1 month has passed since it was built. In addition, there are few reports of intraperitoneal placement of a gastrostomy tube once the tract is mature [7]. Therefore, we selected LAC as the surgical procedure. First, we observed the abdominal cavity under low insufflation pressure to confirm that the fistula was robust. Thereafter, the insufflation pressure was gradually increased. Although no data existed regarding the appropriate insufflation pressure for patients with PEG, 10 mmHg seemed to be safe under cautious observation. There was no problem concerning gastrostomy during surgery in this case. One easy way to determine the presence of damage to the fistula is to check the PEG. If the fistula is compromised during the LAC, gas will leak. In some cases, subcutaneous emphysema may appear. At the same time, it is very important to be careful not to touch the fistula during surgery, because the fistula is extended and gets thinner.

Patients with gastrostoma are usually elderly or have lower levels of activities of daily living. For these patients, less invasive surgery is required.

The number of LAC procedures carried out for colorectal cancer is increasing in Japan. LAC has been proven to be comparable oncologically to open colectomy by prospective randomized controlled studies [8-13]. In addition, the LAC group had shorter hospital stay durations and less analgesic use [8]. Although the operative time was significantly longer in the LAC group [8], LAC can be an option for treatment of patients with colorectal cancer after PEG who require minimally invasive surgery.

There are potential limitations in terms of surgical treatment for patients with colorectal cancer after PEG. First, the surgical treatment for colorectal cancer may be a contraindication for some patients, such as those with terminal conditions. Second, laparoscopic surgery cannot be performed on some patients with cardiopulmonary dysfunction. If the patients do not have any contraindications, LAC can be a potential option for the treatment of colorectal cancer in patients after PEG.

## Conclusion

In conclusion, carefully performed laparoscopic colectomy is a potential option for the treatment of colorectal cancer in patients with PEG if the PEG tract is mature.

## Consent

Written informed consent was obtained from the patient for publication of this case report and any accompanying images. A copy of the written consent is available for review from the Editor-in-Chief of this journal.

## Competing interest

The authors declared that they have no competing interests.

## Authors’ contributions

HK wrote the main manuscript and performed the operation. All authors read and approved the final manuscript.
